# Prevalence and predictors of common psychoactive substances consumption among university students: the role of mental health and academic fields

**DOI:** 10.3389/fpsyt.2025.1658600

**Published:** 2025-10-08

**Authors:** Eleonora Espinoza-Turcios, Lysien Ivania Zambrano, Henry Noel Castro-Ramos, Carlos Antonio Sosa-Mendoza, Itzel Carolina Fuentes-Barahona, Fausto Muñoz-Lara, Altay Alves Lino de Sousa

**Affiliations:** ^1^ Department of Public Health, Faculty of Medical Sciences, National Autonomous University of Honduras (UNAH), Tegucigalpa, Honduras; ^2^ Group GRINVAR, Faculty of Medical Sciences (FCM), National Autonomous University of Honduras (UNAH), Tegucigalpa, Honduras; ^3^ Mental Health Research Group, Faculty of Medical Sciences (FCM), National Autonomous University of Honduras (UNAH), Tegucigalpa, Honduras; ^4^ Department of Morphological Sciences, Faculty of Medical Sciences (FCM), National Autonomous University of Honduras (UNAH), Tegucigalpa, Honduras; ^5^ Honduran Institute for the Prevention of Alcoholism, Drug Addiction and Drug Dependency (IHADFA), Tegucigalpa, Honduras; ^6^ Department of Gynecology, Faculty of Medical Sciences (FCM), National Autonomous University of Honduras (UNAH), Tegucigalpa, Honduras; ^7^ Department of Internal Medicine, Faculty of Medical Sciences (FCM), National Autonomous University of Honduras (UNAH), Tegucigalpa, Honduras; ^8^ Department of Internal Medicine, Teaching Hospital, Tegucigalpa, Honduras; ^9^ Psychobiology Department, Federal University of Sao Paulo, São Paulo, Brazil

**Keywords:** gender and health, fatigue, mental health, caffeine, students, drug users

## Abstract

**Aim:**

This research focuses on to identify the key predictors of consumption of caffeinated beverages (coffee, tea, and energy drinks) among Honduran university students, focusing on factors such as gender, fatigue, and academic discipline.

**Methods:**

A cross-sectional analytical study was conducted at the National Autonomous University of Honduras (UNAH), with students from different programs and academic years enrolled in the second semester of 2023 and the first semester of 2024. The sample consisted of 1,181 students from various faculties, was carried out through surveys in the classroom, before or after classes, ensuring the voluntary participation of students. Participants completed an anonymous Microsoft Forms survey including validated instruments to assess insomnia (severity of insomnia), fatigue (Chalder Fatigue Questionnaire), daytime sleepiness (Epworth Sleepiness Scale), and symptoms of depression, anxiety, and stress (DASS-21). Regression models were used to verify the associated factors with use of consumption of caffeinated beverages in past 6 months and use of illicit drugs at least once in life (Logistic regression) and for the number of different consumptions of caffeinated used in last 6 months, a Poisson model was build.

**Results:**

83.7% of participants consumed coffee, and 46.8% consumed energy drinks. Men were 3.2 times more likely to consume caffeinated beverages than women. Fatigue was associated with a higher likelihood of consuming energy drinks (OR = 2.10, 95% CI: 1.24–3.55). Economics students were 2.15 times more likely to consume caffeinated beverages, compared to Medical Sciences students (OR = 2.15, 95% CI: 1.06–4.33).

**Conclusions:**

Factors such as male sex fatigue, and academic discipline, especially Economics, are significant predictors of caffeinated beverages consumption. Specific interventions are needed to mitigate the negative effects on students mental and physical health.

## Introduction

The consumption of stimulants of both legal and illegal drugs has become a global public health problem among young people (1). The causes are multifactorial, including social context, drug availability, and personal characteristics. The World Health Organization (WHO) highlights that 2.6 million deaths per year were attributable to alcohol consumption (2 million men) and 0.6 million deaths to psychoactive drug use (0.4 million men) (2).

The 2023 Global Burden of Disease (GBD) study (3), suggests that the burden from substance use increases substantially during adolescence and early adulthood. While illicit drugs cause a greater burden in the U.S., Canada, Australia, New Zealand, and Western Europe. Stress is a notable risk factor, as addictions result from biological, environmental, and drug-induced interactions (4). In 2017, approximately 271 million people (5.5% of the global population aged 15–64) used drugs, marijuana was the most consumed illicit drug (188 million users) (5).

Caffeinated beverages, enhance mental alertness, attention, and energy. In Latin America, El Salvador had the highest stimulant use (0.7%), followed by Ecuador (0.6%), with higher rates among men—except in Panama, where women exceeded men. Cannabis consumption in 2019 reached 3.3% in El Salvador and Panama, with the lowest rates in Venezuela, Bolivia, and Peru (around 3.1%) (6).

The consumption of energy drinks alongside other substances has also grown. According to the 2020 EDADES survey (Spain), 12.3% of the population aged 15–64 consumed energy drinks in the past 30 days—15.3% of men and 9.1% of women. Consumption was highest among individuals aged 15–24, with a 32.2% prevalence (38.3% for men and 25.7% for women) (7).

Temple JL (2019), conducted a review of the National Library of Medicine database on the safety of caffeine consumption in children and adolescents, finding that typical, moderate consumption is relatively safe in these groups. Consuming high doses of caffeine (>400 mg) can cause physiological, psychological and behavioral harm, children with psychiatric or cardiac conditions are at greater risk (8). In Honduras, Buchanan and Pillon (2008) reported that university students mainly consumed caffeine, mate, energy drinks, and Coca-Cola, and to a lesser extent, marijuana, cocaine, Valium, and sleep aids. Women consumed these substances to combat fatigue and improve academic performance; men, to have fun and relieve stress (9).

Licona Rivera et al. (2016), in a cross-sectional descriptive study in Honduras where 278 students participated, found that 80 students (29%) used drugs: alcohol (48.8%), energy drinks (16.3%), and marijuana (10%). Among medical students, 34% of them reported consumption, followed by 24% of dental and 22% of nursing students (10). In a sample of 1,510 (77%) Honduran students reported using stimulants during the academic year, with a female majority (55%). The age group under 25 accounted for 95% of the reported use (11).

Previous research has identified several predictive factors for stimulant use among university students, including male gender, higher levels of fatigue, specific academic disciplines with heavier workloads, and elevated stress or anxiety levels. These factors influence consumption patterns through mechanisms such as the perceived need for improved concentration, enhanced alertness, and coping with academic pressure. Understanding these predictors is essential for developing targeted health interventions and for interpreting the variation in consumption across different student populations.

Despite the growing literature on substance use among university students worldwide, there is a lack of recent, comprehensive data from Honduras that examines both caffeinated beverage consumption and other psychoactive substances across different academic disciplines. Previous local studies have either focused on small samples or limited types of substances, hindering the ability to develop tailored prevention strategies. Our study addresses this gap by providing updated epidemiological data from a large, stratified sample of university students, while also identifying sociodemographic and psychosocial predictors of consumption. The objective of this research is to assess the prevalence and predictors of caffeinated beverage and other psychoactive substance use among Honduran university students, with the aim of informing targeted interventions and institutional policies that promote healthier lifestyles and reduce substance-related risks.

## Methods

### Study design

Study location: The study was conducted at the National Autonomous University of Honduras (UNAH), in the university city of Tegucigalpa, with students from different programs and academic years enrolled in the second semester of 2023 and the first semester of 2024.

Sample size calculation: The sample size was calculated based in the Kish Coefficient A measure of within-stratum variance, indicating how much sample size is needed related with a specific amount of variance in optimal allocation to stratified sampling. In the Universidad Nacional Autonoma de Honduras (UNAH) we have 36,037 students; with 5% confidence level and estimated precision error of 3%, a sample of 1037 students, allocated proportionally among the UNAH Departments, it is possible to assess the prevalences of Drug consumption and caffeinated beverages, for the majority of UNAH Departments. Sampling method: A probabilistic sampling was carried out by strata.

The inclusion criteria were being an enrolled student at UNAH, aged ≥18 years, having an active registration in the second semester of 2023 and the first semester of 2024, and possessing a valid student ID number (CI). The exclusion criteria were not having a registered email address, being unable to provide the requested information, and being an inactive student during the study period. Type of study: A cross-sectional and analytical study.

With a sample of 1,037 participants, using stratified probabilistic sampling by faculty: Humanities and Arts (87); Social Sciences (116); Sciences (80); Economics (457); Law (83); Medical Sciences (66); Chemical and Pharmaceutical Sciences (18); Engineering (119); Dentistry (3); Space Sciences (9), ensuring a representative distribution of participants across different disciplines.

The study was approved by the Biomedical Research Ethics Committee of the Faculty of Medical Sciences (CEIB), approval code 067-2023, dated October 20, 2023. The ethical principles of the 2013 Helsinki Declaration were respected, ensuring confidentiality and data protection.

The study was carried out through surveys in the classroom, before or after classes, ensuring the voluntary participation of students. The research team coordinated with faculty members from different programs to explain the objectives of the study and schedule specific time slots for recruitment. Students received a detailed presentation about the study, and those willing to participate provided their written informed consent (two copies: one for the participant and another for the research team). Each participant received a QR code linking them to an anonymous Microsoft Forms survey, allowing them to complete the questionnaire on-site or when convenient later.

### Measures

The following scales were used: Insomnia Severity Index (ISI): Measures the perceived severity of insomnia, focusing on the level of sleep pattern disruption, the consequences of insomnia, and the degree of concern and distress related to sleep problems ((0–7: No clinically significant insomnia; 8–14: Subthreshold insomnia; 15–21: Moderate insomnia; 22–28: Severe insomnia)) (12), It has been validated in Spanish and there is a validation in Mexican adults (13). Chalder Fatigue Questionnaire (CFQ): Measures physical and mental fatigue in patients with chronic fatigue syndrome, followed by correlations with subjective and objective cognitive performance measures, physiological strength and work capacity measures, depression, anxiety, and subjective sleep difficulties. Chalder Fatigue scores ≥4 were classified as “Yes” (presence of significant fatigue) and <4 as “No,” following published cut-offs validated in Spanish (14).

Epworth Sleepiness Scale (ESE): An instrument used to quantify daytime sleepiness, already validated in Chile, Scale scores ≥10 were classified as “Yes” (excessive daytime sleepiness) and <10 as “No,” (0–5: Lower normal daytime sleepiness; 6–10: Higher normal daytime sleepiness; 11–12: Mild excessive daytime sleepiness; 13–15: Moderate excessive daytime sleepiness; 16–24: Severe excessive daytime sleepiness) (15).

DASS-21: Contains three subscales: Depression (Normal (0–9), Mild (10–13), Moderate (14–20), Severe (21–27), Extremely severe (28+)), Anxiety (Normal (0–7), Mild (8–9), Moderate (10–14), Severe (15–19), Extremely severe (20+), and Stress (Normal (0–14), Mild (15–18), Moderate (19–25), Severe (26–33), Extremely severe (34+). Scales validated in Chilean university students. Construct validity was verified through exploratory factor analysis, confirming the adequate reliability and internal consistency of DASS-21 (16).

### Statistical analysis

All statistical analyses were conducted with JAMOVI. Data cleaning, coding, and validation were carried out before the analysis to ensure integrity and accuracy. For our binary outcomes (Use of caffeinated beverages, in past 6 months and Use of Illicit Drugs (Hallucinogens Benzodiazepines, Cocaine, Crack, Marijuana at least once in life), logistic regression models with Wald Method were applied to estimate the odds ratios (OR) and their corresponding 95% confidence intervals (CI), evaluating the factors associated with the consumption of energy drinks and drug use over the lifetime. For our count outcome (Number of Different caffeinated beverages, used in last 6 months), a Poisson regression model was used (Generalized Linear Model using REML estimation method) to examine the factors influencing the number of caffeinated beverages consumed in the last six months, with rate ratios (RR) and 95% CIs calculated according. For all analysis, a significant level of 5% (p<0.05) was considered.

## Results

A total of 1,181 participants were obtained reach, with 989 (83.7%) declaring coffee consuming 553 (46.8%) energy drinks, and 395 (33.4%) tea. Regarding alcohol consumption, 208 (17.6%) participants consumed alcohol, 107 (9.1%) used tobacco, and 28 (2.4%) used other drugs. Regarding faculties and consumption, the Faculty of Humanities and Arts reported the highest coffee consumption at 85 (89.5%), followed by energy drinks and tea, with the Faculty of Dentistry reporting 4 (80%) and 3 (60%) respectively. Alcohol consumption was 27 (25.7%) and drug consumption 6 (5.7%) was concentrated in the Faculty of Law, while tobacco consumption was highest in the Faculty of Space Sciences with 3 (25%). The results are presented in **Table 1**.

**Table 1:** Prevalence of stimulants and drugs use among Honduran university students, by Department.

Department	Coffee				Energetics				Tea				Alcohol				Tobacco				Other drugs				Total
	No		Yes		No		Yes		No		Yes		No		Yes		No		Yes		No		Yes		
	N	%	N	%	N	%	N	%	N	%	N	%	N	%	N	%	N	%	N	%	N	%	N	%	N
Dentistry	1	20	4	80	1	20	4	80	2	40	3	60	5	100	0	0	5	100	0	0	5	100	0	0	30
Engineering	23	20	95	80	59	50	59	50	89	75	29	25	100	85	18	15	111	94	7	6	118	100	0	0	708
Humanities	10	11	85	89	50	53	45	47	62	65	33	35	77	81	18	19	84	88	11	12	91	96	4	4	570
SocialSci	16	12	116	88	76	58	56	42	91	69	41	31	109	83	23	17	114	86	18	14	129	98	3	2	792
ChemSciPharm	3	14	19	86	11	50	11	50	15	68	7	32	19	86	3	14	20	91	2	9	22	100	0	0	132
MedicalSci	17	21	66	79	50	60	33	40	54	65	29	35	66	80	17	20	73	88	10	12	79	95	4	5	498
LegalSci	21	20	84	80	59	56	46	44	69	66	36	34	78	74	27	26	90	86	15	14	99	94	6	6	630
SpaceSci	3	25	9	75	5	42	7	58	8	67	4	33	9	75	3	25	9	75	3	25	12	100	0	0	72
EconomicSci	78	17	394	83	236	50	236	50	311	66	161	34	403	85	69	15	444	94	28	6	465	98	7	2	2832
Science	20	15	117	85	81	59	56	41	85	62	52	38	107	78	30	22	124	90	13	10	133	97	4	3	822
Total	192		989		628		553		786		395		973		208		1074		107		1153		28		

The binary logistic regression model identified sex, fatigue (Chalder scale), and faculty of study as significant predictors of energy drink consumption in the last six months: men were 3.2 times more likely to consume energy drinks compared to women (OR = 3.21, 95% CI: 1.91–5.39, p < 0.001). Higher levels of fatigue were associated with a greater likelihood of consuming energy drinks (OR = 2.10, 95% CI: 1.24–3.55, p = 0.006). Students enrolled in Economics were 2.15 times more likely to consume energy drinks compared to those in Medical Sciences (OR = 2.15, 95% CI: 1.06–4.33, p = 0.033). Other factors, such as marital status, age, stress, anxiety, and other groups of professors, showed no statistically significant associations (p > 0.05). The results are presented in **Table 2**.

**Table 2:** Binary logistic regression with the associated variables with the psychoactive substances use in the past 6 months.

	Use of psychoactive substances							95% Confidence Interval	
Predictor	No	Yes	Estimate	SE	Z	p	Odds ratio	Lower	Upper
Intercept	% (n/total)	% (n/total)	0.5	0.94	0.53	0.6	1.65	0.26	10.39
Marital status									
Single (ref) - Not Married	0.9 98/110	0.9 1005/1071	0.79	0.43	1.84	0.07	2.2	0.95	5.08
Married (ref) - Not Married	0.0 4/110	0.0 26/1071	0.72	0.71	1.01	0.31	2.05	0.51	8.2
Sex									
Male (ref) - Female	0.2 25/110	0.4 449/1068	1.17	0.26	4.4	<.001	3.21	1.91	5.39
Age in years	19 (21-24)	19 (21-23)	0	0	0.32	0.75	1	0.99	1.01
Stress									
Yes (ref) - No	0.4 40/110	0.3 319/1071	-0.37	0.24	-1.5	0.13	0.69	0.43	1.11
Anxiety									
Yes (ref) - No	0.8 89/110	0.8 863/1071	-0.05	0.28	-0.2	0.86	0.95	0.54	1.66
Epworth									
Yes (ref) - No	0.3 35/110	0.3 324/1071	-0.13	0.23	-0.5	0.59	0.88	0.56	1.39
Chalder									
Yes (ref) - No	0.3 30/110	0.4 468/1071	0.74	0.27	2.77	0.01	2.1	1.24	3.55
ISI									
Yes (ref) - No	0.6 63/110	0.4 415/1071	0.41	0.26	1.59	0.11	1.51	0.91	2.51
Department									
Engineering (ref) - Medical Sciences	0.1 14/110	0.1 104/1071	-0.21	0.45	-0.5	0.64	0.81	0.33	1.95
Sciences (ref) - Medical Sciences	0.1 14/110	0.1 123/1071	0.33	0.43	0.77	0.44	1.4	0.6	3.26
Legal Sciences (ref) - Medical Sciences	0.1 9/110	0.1 96/1071	0.62	0.48	1.31	0.19	1.87	0.74	4.74
Space Sciences (ref) - Medical Sciences	0.0 1/110	0.0 11/1071	0.03	1.11	0.03	0.98	1.03	0.12	9.15
Economic Sciences (ref) - Medical Sciences	0.4 39/110	0.4 433/1071	0.76	0.36	2.13	0.03	2.15	1.06	4.33
Humanities and Arts (ref) - Medical Sciences	0.0 5/110	0.1 90/1071	0.8	0.57	1.4	0.16	2.22	0.73	6.76
Social Sciences (ref) - Medical Sciences	0.1 11/110	0.1 121/1071	0.8	0.45	1.79	0.07	2.22	0.93	5.34
Chemical and Pharmaceutical Sciences (ref) - Medical Sciences	0.0 3/110	0.0 19/1071	0.09	0.72	0.12	0.91	1.09	0.26	4.49
Dentistry (ref) - Medical Sciences	0.0 0/110	0.0 5/1071	13.6	635	0.02	0.98	8E+05	0	Inf
Drug Use Once in Life									
Yes (ref) - No	0.1 13/100	0.2 227/1071	0.49	0.32	1.52	0.13	1.63	0.87	3.04

*1.* Estimates represent the log odds of " Do you consume Stimulant Beverages in the last 6- months = Yes" vs. " Do you consume Stimulant Beverages in the last 6- months = No". *2.* Cutoff points for the Psychometric Scales: ISI (Yes > 8 points); Chalder (CFQ Yes > 4 points); Epworth Scale (ESS > 11 points); DASS-21 Scale (Anxiety > 7 points, Stress > 15 points)

An independent binary logistic regression model evaluated the predictors of lifetime drug use and revealed the following: Men were 2.6 times more likely to use drugs over their lifetime compared to women (OR = 2.62; 95% CI: 1.91–3.59; p < 0.001). Stress was significantly associated with lifetime drug use (OR = 1.58; 95% CI: 1.13–2.22; p = 0.008). Engineering students were significantly less likely to report lifetime drug use compared to medical sciences students (OR = 0.47; 95%CI: 0.22–0.97; p = 0.040). Other predictors, such as marital status, anxiety, insomnia, fatigue (Chalder scale), and energy drink consumption, showed no statistically significant associations with lifetime drug use (p > 0.05). The results are presented in **Table 3**.

**Table 3:** Binary logistic regression with the associated variables with the drug consumption once in life.

	Drug Consumption once in life							95% Confidence Interval	
Predictor	No	Yes	Estimate	SE	Z	p	Odds ratio	Lower	Upper
Intercept	% (n/total)	% (n/total)	-2.42	0.55	-4.41	<.001	0.09	0.03	0.26
You have consumed energy drinks in the last 6 months Coffee									
Yes - No	0.9 844/941	0.9 227/240	0.48	0.32	1.5	0.134	1.61	0.86	3
Department									
Engineering (ref) - Medical Sciences	0.1 97/941	0.1 21/240	-0.77	0.37	-2.06	0.04	0.47	0.22	0.97
Sciences (ref) - Medical Sciences	0.1 100/941	0.2 37/240	0.1	0.34	0.29	0.772	1.1	0.57	2.15
Legal Sciences (ref) - Medical Sciences	0.1 77/941	0.1 28/240	0.14	0.36	0.38	0.702	1.15	0.57	2.3
Space Sciences (ref) - Medical Sciences	0.0 7/941	0.0 5/240	0.59	0.67	0.88	0.381	1.8	0.48	6.72
Economic Sciences (ref) - Medical Sciences	0.4 397/941	0.3 75/240	-0.51	0.3	-1.7	0.09	0.6	0.33	1.08
Humanities and Arts (ref) - Medical Sciences	0.1 74/941	0.1 21/240	-0.35	0.38	-0.93	0.354	0.71	0.34	1.48
Social Sciences (ref) - Medical Sciences	0.1 101/941	0.1 31/240	0.02	0.35	0.06	0.953	1.02	0.52	2.01
Chemical and Pharmaceutical Sciences (ref) - Medical Sciences	0.0 19/941	0.0 3/240	-0.65	0.69	-0.94	0.345	0.52	0.13	2.02
Dentistry (ref) - Medical Sciences	0.0 5/941	0.0 0/240	-13.27	385.38	-0.03	0.973	0	0	0.05
Civil status									
Single (ref) – Not Married	0.9 880/941	0.9 223/240	-0.07	0.37	-0.19	0.852	0.93	0.46	1.92
Married (ref) – Not Married	0.0 24/941	0.0 6/240	0.05	0.6	0.09	0.928	1.06	0.33	3.42
Sex									
Male (ref) - Female	0.4 339/939	0.6 135/239	0.96	0.16	5.98	<.001	2.62	1.91	3.59
Age (years)	19.0 (21.0-23.0)	20.0 (21.0-23.6)	0	0	-0.19	0.853	1	0.99	1.01
Stress									
Yes (ref) - No	0.3 266/941	0.4 93/240	0.46	0.17	2.66	0.008	1.58	1.13	2.22
Anxiety									
Yes (ref) - No	0.8 750/941	0.8 202/240	0.07	0.21	0.33	0.743	1.07	0.7	1.63
Epworth									
Yes (ref) - No	0.3 278/941	0.3 81/240	0.01	0.17	0.04	0.965	1.01	0.72	1.4
Chalder									
Yes (ref) - No	0.4 382/941	0.5 116/240	0.26	0.17	1.49	0.137	1.29	0.92	1.82
ISI									
Yes (ref) - No	0.4 400/941	0.3 78/240	0.31	0.18	1.75	0.08	1.36	0.96	1.92

1. Estimates represent the log odds of "Have you ever used any drugs in life = Yes" vs. "Have you ever used any drugs in life = No". 2. Cutoff points for the Psychometric Scales: ISI (Yes > 8 points); Chalder (CFQ Yes > 4 points); Epworth Scale (ESS > 11 points); DASS-21 Scale (Anxiety > 7 points, Stress > 15 points)

A Poisson regression model was used to evaluate the predictors of caffeinated beverages consumption rates during the last six months: Men had a 57% higher consumption rate of caffeinated beverages than women (RR = 1.57; 95% CI: 1.47–1.68; p < 0.001). Fatigue (Chalder scale) was associated with a 17% higher caffeinated beverages consumption rate (RR = 1.17; 95% CI: 1.09–1.25; p < 0.001). Anxiety was also a significant predictor (RR = 1.18; 95% CI: 1.08–1.28; p < 0.001).

Students from Economics (RR = 1.13; 95% CI: 1.01–1.26; p = 0.029) and Humanities and Arts (RR = 1.17; 95% CI: 1.02–1.35; p = 0.027) had significantly higher rates of caffeinated beverages consumption compared to students from Engineering. Greater severity of insomnia (ISI scale) was associated with a 19% lower caffeinated beverages consumption rate (RR = 0.81; 95% CI: 0.75–0.87; p < 0.001). Other predictors, including stress (p = 0.052), marital status, and other groups of professors, were not statistically significant. The results are presented in **Table 4**.

**Table 4:** Poisson regression with the associated factors for the number of different stimulants consumed in the last 6 months.

					95% Exp(B) Confidence Interval			
Names	Effect	Estimate	SE	exp(B)	Lower	Upper	z	p
(Intercept)	(Intercept)	1.09	0.06	2.98	2.65	3.33	18.77	<.001
Chalder	Yes (ref) - No	0.15	0.04	1.17	1.09	1.25	4.25	<.001
Epworth	Yes (ref) - No	-0.03	0.04	0.97	0.90	1.04	-0.86	0.391
Anxiety	Yes (ref) - No	0.16	0.04	1.18	1.08	1.28	3.60	<.001
Stress	Yes (ref) - No	-0.07	0.04	0.93	0.86	1.00	-1.94	0.052
Sex	Male (ref) - Female	0.45	0.03	1.57	1.47	1.68	13.49	<.001
Age (years)	Age in years	0.00	0.00	1.00	1.00	1.00	1.77	0.077
Department	Science (ref) - Engineering	0.08	0.07	1.08	0.94	1.24	1.11	0.267
	Medical Sciences (ref) - Engineering	0.12	0.08	1.12	0.96	1.31	1.47	0.142
	Legal Sciences (ref) - Engineering	0.06	0.07	1.06	0.92	1.23	0.78	0.434
	Space Sciences (ref) - Engineering	-0.06	0.16	0.94	0.67	1.28	-0.37	0.711
	Economic Sciences (ref) - Engineering	0.12	0.06	1.13	1.01	1.26	2.19	0.029
	Humanities and Arts (ref) - Engineering	0.16	0.07	1.17	1.02	1.35	2.21	0.027
	Social Sciences (ref) - Engineering	0.13	0.07	1.14	0.99	1.31	1.81	0.070
	Chemical Sciences and Pharmacy (ref) - Engineering	0.14	0.13	1.16	0.89	1.47	1.14	0.256
	Dentistry (ref) - Engineering	0.18	0.25	1.19	0.70	1.88	0.71	0.477
Marital Status	Single (ref) - Unmarried	0.16	0.09	1.17	1.00	1.39	1.87	0.061
	Married (ref) - Unmarried	0.15	0.13	1.16	0.89	1.51	1.12	0.262
ISI	No Insomnia (ref) - Insomnia	-0.21	0.04	0.81	0.75	0.87	-5.74	<.001

## Discussion

The objective of the study was to identify the key predictors of consumption of caffeinated beverages (coffee, tea, and energy drinks) and drug use among Honduran university students. Some isolated studies have analyzed university populations by faculty, particularly in Medical and Dental Sciences. According to the International Classification of Diseases, stimulants are substances that alter the functions of the central nervous system. Common examples include cocaine, amphetamines, methamphetamines, and MDMA (3,4-methylenedioxy-methamphetamine) (17). Energy drinks are also considered stimulants due to their high caffeine content, which acts on the CNS to improve physical and cognitive performance. They also typically include taurine, B vitamins, and sugars (18).

A systematic review identified coffee as the most used stimulant among medical students, followed by tea and other forms of caffeine, including energy drinks. These substances are popular because of the belief that they can improve cognitive function quickly and with little effort. Caffeine is consumed by 80% of the global population for its psychoactive effects, and almost 89% in the United States (19).

The study found that coffee was the most consumed caffeinated beverage, followed by energy drinks and tea findings consistent with previous studies like that of Chávez-Gutiérrez J. In our sample, 876 students (58%) consumed coffee, 453 (30%) consumed energy drinks. Notably, 378 students (25%) were unaware of the potential adverse effects, while 1,132 (75%) reported being aware of the risks but continued consumption (11).

Among Honduran university students, 208 (17.6%) consumed alcohol, 107 (9.1%) tobacco, and 28 (2.4%) used other illicit drugs. A cross-sectional study with 325 students from the Faculty of Chemistry and Pharmacy at the National Autonomous University of Honduras revealed that 99 students (30.5%) reported alcohol use, and 29 (8.9%) reported marijuana use (20).

Differences by academic discipline showed that Economics students had significantly higher odds of caffeinated beverages use (OR = 2.15), while Engineering students showed lower odds of lifetime drug use (OR = 0.47). However, further detailed analysis is required to understand how academic workload and environmental factors influence substance use. A study conducted among university students in Tabasco, Mexico, reported increased energy drink consumption during exam periods, indicating specific stress-related risk factors in this population (21).

The consumption of high doses of caffeine has been associated with several health disorders, including sleep disturbances, anxiety, and caffeine toxicity (22–24). In this study, significant predictors of Differences by academic discipline showed that Economics students had significantly higher odds of caffeinated beverages use included male gender, fatigue measured by the Chalder scale, and being enrolled in the Faculty of Economics. A related study at Universidad Latina de Costa Rica showed no significant influence of sex, marital status, employment, or faculty on stimulant use—except for a noted association between nicotine use and sex (25).

Fatigue and anxiety emerged as strong predictors of caffeinated beverages consumption. Higher fatigue levels measured via the Chalder scale, combined with anxiety symptoms, were associated with increased use. Stress also showed a significant correlation with drug use (OR = 1.58; p = 0.008). Risk behaviors such as combining energy drinks with alcohol, tobacco, or illicit drugs were linked to poorer health outcomes, diminished quality of life, and mental health problems. A cohort analysis from the U.S. Nurses’ Health Study 3, involving over 46,000 participants, found high comorbidity between energy drink consumption and use of marijuana, tobacco, alcohol, and other drugs (26).

Fatigue is recognized as a predictor of general and mental health in different populations, affecting quality of life, sleep quality, and emotional well-being (27). Stress and fatigue significantly impact students’ academic success and social adaptability (28). Fatigue emerged as another key predictor in the study, suggesting that students may rely on caffeine as a compensatory strategy to manage academic workload and irregular sleep patterns.

Luneke A. et al. found in a U.S. university study that energy level enhancement and anxiety-related effects were major predictors of energy drink us (29). Similarly, a systematic review and meta-analysis by Nadeem IM et al. showed the top reasons for consuming energy drinks were boosting energy/reducing fatigue (24.5%), staying awake (15.7%), and improving study concentration (14.1%) (30).

In the study, it was found that men consume more drugs over their lifetime compared to women, male students are strongly associated with higher consumption of caffeinated beverages, which may be explained by greater engagement in social behaviors involving such drinks, as well as lower perceived health risks compared to female students. Stress was significantly associated with lifetime drug consumption. Engineering students had a significantly lower probability of reporting lifetime drug use compared to medical sciences students.

Our findings also revealed that men reported higher lifetime drug use compared to women, and stress was strongly associated with that use. Engineering students were significantly less likely to report lifetime drug use compared to students from enrolled in Medical Sciences, could reflect differences in curriculum structure, assessment schedules, and stress management resources available within faculties. These findings suggest that academic environment and field of study play important roles in consumption patterns. Stress linked to academic pressure, lifestyle changes, clinical practice, and the lack of supportive or preventive mechanisms from the educational system can negatively affect students’ mental health and overall education quality (31, 32).

Other demographic and psychosocial variables such as marital status, anxiety, insomnia, and fatigue (as measured by the Chalder scale) did not show statistically significant associations with lifetime drug use in this study. One notable finding was the greater risk of substance consumption among men compared to women (OR = 3.21; OR = 2.62), particularly among males from lower socioeconomic backgrounds (33).

This was consistent with international studies. For instance, research in Italy showed higher prevalence among male university students, often associated with risk behaviors like smoking and alcohol use. These results align with data from the 2018 NaSSDA (National Secondary Students Diet and Activity) survey, which reported that 16% of students consumed at least one energy drink per week (34–36).

Students enrolled in Economics and Humanities and Arts reported significantly higher rates of caffeinated beverages consumption compared to Engineering students. Higher insomnia levels, measured using the ISI scale, were also associated with increased caffeinated beverages use. A particularly concerning trend is the combined use of caffeinated beverages and drugs at social events, such as parties. The cardiovascular effects of caffeine such as elevated heart rate and blood pressure can be exacerbated when combined with alcohol and may impair the perception of intoxication. This interaction increases the risk of overconsumption and acute complications (37).

Our findings align with previous reports indicating higher caffeine consumption among male university students and an association between fatigue and energy drink intake (25, 34, 35), we observed a significant effect of academic discipline, with Economics students showing higher consumption rates than those in Medical Sciences. These differences may reflect variation in academic workload, cultural norms, or access to caffeinated products across faculties. Future research should investigate causal mechanisms and the role of psychosocial stressors in shaping consumption patterns.

The findings of this study contribute to the theoretical understanding of substance use behaviors in university settings by highlighting the interplay between individual factors (e.g., gender, fatigue, stress) and contextual elements (e.g., faculty environment) in shaping consumption patterns. From a practical standpoint, these results support the development of targeted health promotion programs that address both behavioral risk factors and institutional conditions that may encourage caffeine and other psychoactive substance use. Interventions could integrate sleep hygiene education, time management skills, and faculty specific stress reduction initiatives. In terms of policy, universities and health authorities could use these data to inform regulations on the availability and marketing of caffeinated products within campuses, as well as to strengthen mental health services aimed at reducing stress related substance use.

### Limitations

This study has several limitations. First, it was conducted at a single public university in the capital city, which may limit the generalizability of the results to other institutions or non-university populations in Honduras. Second, the cross-sectional design prevents establishing causality between predictors and substance use. Third, the data relied on self-reported measures for caffeine and other substance consumption, which may be subject to recall bias and social desirability bias, potentially leading to underreporting or overreporting of actual use. Fourth, the study did not include certain stimulant categories such as prescription or illicit amphetamines, focusing instead on common psychoactive substances, which may limit comparability with broader stimulant use research, limited statistical power in certain subgroups, there are small categories which limit their interpretation Additionally, differences in academic calendars, workload, and extracurricular culture across faculties may have influenced consumption patterns but were not systematically measured. Finally, although validated instruments were used, residual confounding from unmeasured psychosocial or environmental factors cannot be ruled out.

## Conclusions

"This study highlights the significant relationship between the male sex, fatigue, and academic discipline as factors associated with caffeinated beverages (coffee, energy drinks) use among Honduran university students, emphasizing the need for specific interventions for this group, especially in faculties such as Economics, where a higher prevalence of consumption is observed. Fatigue, as measured by the Chalder Scale, suggests that students experiencing high levels of fatigue are more likely to turn to stimulants to improve academic performance and combat exhaustion.

It is necessary to re-evaluate the availability and accessibility of caffeinated beverages (coffee, energy drinks) in educational institutions. The implementation of educational programs about the risks of excessive consumption of these substances, along with mental health interventions focused on preventing fatigue and stress management, could mitigate the observed negative effects. Further longitudinal studies are warranted to assess causal relationships and evaluate the effectiveness of preventive measures.

## Data Availability

The datasets presented in this study can be found in online repositories. The names of the repository/repositories and accession number(s) can be found below: https://papers.ssrn.com/sol3/papers.cfm?abstract_id=5245857.
